# Longitudinal assessment of classic and 11-oxygenated androgen concentrations and their association with type 2 diabetes mellitus development: the Tromsø study

**DOI:** 10.1007/s00592-024-02266-5

**Published:** 2024-03-18

**Authors:** Giovanni Allaoui, Charlotta Rylander, Ole-Martin Fuskevåg, Guri Grimnes, Maria Averina, Tom Wilsgaard, Vivian Berg

**Affiliations:** 1 Division of Diagnostic Services, Department of Laboratory Medicine, University Hospital of North-Norway, 9038 Tromsø, Norway; 2 Department of Medical Biology, Faculty of Health Sciences, UiT-The Arctic University of Norway, 9037 Tromsø, Norway; 3 Department of Community Medicine, Faculty of Health Sciences, UIT-The Arctic University of Norway, 9037 Tromsø, Norway; 4 Department of Clinical Medicine, Faculty of Health Sciences, UIT-The Arctic University of Norway, 9037 Tromsø, Norway; 5 Division of Medicine, University Hospital of North-Norway, 9038 Tromsø, Norway

**Keywords:** Androgens, Type 2 diabetes mellitus, Longitudinal survey, Preventive, Health service

## Abstract

**Aim:**

We aimed to investigate changes in pre-diagnostic concentrations of classic and 11-oxygenated androgens in type 2 diabetes (T2DM) cases and healthy controls, associations between androgen concentrations and T2DM, and the potential for androgens to improve the prediction of T2DM when considered in combination with established risk factors.

**Methods:**

Androgen concentrations were analysed in serum samples from 116 T2DM cases and 138 controls at 3, pre-diagnostic time-points: 1986/87 (T1), 1994/95 (T2), and 2001 (T3). Generalised estimating equations were used to longitudinally examine androgen concentrations, and logistic regression models were used to estimate the odds ratios (OR) of T2DM at each time-point. Logistic regression models were also used to calculate area under the receiver operating characteristics curve (AROC) from models including established risk factors alone (ERF model) and established risk factors plus each androgen, respectively, which were compared to identify improvements in predictive ability.

**Results:**

For women, no significant associations were observed between any of the investigated androgens and T2DM after adjusting for confounders. For men, after adjusting for confounders, concentrations of all investigated 11-oxygenated androgens were higher in cases than controls at one or several time-points. We observed associations between T2DM and concentrations of 11-ketoandrostenedione (OR: 1.59) and 11-ketotestosterone (OR: 1.62) at T1; and 11-hydroxyandrostenedione (OR: 2.00), 11-hydroxytestosterone (OR: 1.76), 11-ketoandrostenedione (OR: 1.84), 11-ketotestosterone (OR: 1.78) and testosterone (OR: 0.45) at T3 in men. The addition of these androgens (including 11-hydroxytestosterone at T2) to the ERF model resulted in an improved ability to predict T2DM in men (AROC: 0.79–0.82). We did not observe significant differences in changes in androgen concentrations over time between cases and controls in either sex.

**Conclusion:**

Our results demonstrate that testosterone and 11-oxygenated androgens are associated with T2DM in men before diagnosis and may be potential biomarkers in T2DM risk assessment.

**Supplementary Information:**

The online version contains supplementary material available at 10.1007/s00592-024-02266-5.

## Introduction

Disruption of androgen homeostasis has been reported to have a sex–specific association with metabolic dysfunction, insulin resistance, and type 2 diabetes (T2DM) [1, 2]. Androgens are a group of steroid hormones produced in the testes, ovaries, and adrenal glands. They exert their effect by binding to androgen receptors and are integral parts of several processes, including sexual development, body shape, growth, energy expenditure, and modulation of the cardiovascular system (Fig. S1) [3, 4]. It has recently been reported that 11-oxygenated androgens have greater androgenic activity than previously believed, and several observations in diseases where the 11-oxygenated androgens are elevated point towards a potential role in the development of T2DM [5]. For instance, women with polycystic ovarian syndrome (PCOS) often have higher serum levels of classic and 11-oxygenated androgens compared to healthy women and is associated with insulin resistance and increased risk of T2DM [2, 5–7]. Also, individuals with conditions that cause elevated 11-oxygenated androgens due to 21-hydroxylase deficiency (in both men and women), and those with conditions that cause reduced testosterone levels due to hypogonadism in men, have an increased risk of T2DM [8, 9]. Several cross-sectional and longitudinal studies have reported inconsistent associations between classic androgens and T2DM, where dehydroepiandrosterone sulphate (DHEAS) showed non-significant associations with T2DM in men and women in one study [10], while another observed that lower levels of DHEAS in men were significantly associated with T2DM [11]. Further, in some studies, testosterone concentrations were positively associated with the risk of T2DM in women, but inversely associated in men [12–14], whereas other studies did not find any association [15, 16]. However, there is a lack of studies on 11-oxygenated androgens, and it is in our interest to understand how serum concentrations of androgens change over time and their association with T2DM. Therefore, we aimed to investigate; (1) The associations between androgen concentrations and T2DM; (2) Pre-diagnostic changes in concentrations of androgens in T2DM cases and controls; and (3) The potential for androgens to improve the prediction of T2DM when considered in combination with established risk factors.

## Materials and methods

### Study population

The Tromsø Study is a large, ongoing health survey with participants from the Tromsø municipality in Northern Norway and has been described in greater detail elsewhere [17]. Briefly, to date, seven surveys of the Tromsø study (Tromsø1–Tromsø7) have been conducted between 1974 and 2016. Participants attended various physical examinations, answered questionnaires, and donated blood samples for future research. Blood sampling procedures and storage for the Tromsø study surveys has been described in greater detail elsewhere [18]. Briefly, samples were collected in standardised manners by venous puncture at the clinical examinations. Samples were kept at room temperature for 30 min, after which the coagulated samples were centrifuged at 2000 g for 10 min. Aliquots of serum were transferred to secondary plastic sample containers within 1 h and stored at − 70 °C.

The present study has a longitudinal, nested case–control design with repeated measurements from participants who attended Tromsø3 (1986/87), Tromsø4 (1994/95), and Tromsø5 (2001), hereafter referred to as time-points 1 (T1), 2 (T2), and 3 (T3) respectively. Initially we included 145 cases who had serum samples available at all time-points, had a diagnosis of T2DM after T3 (2001) recorded in a local diabetes registry [19], had no other known metabolic diseases, and did not use diabetes medication. We also included 145 controls, who were randomly selected among participants with serum samples available at all time-points and without any T2DM diagnosis at any time-point. The case identification has been described in detail in our previous publications on the same study population [20]. Further, we excluded 29 cases and seven controls with HbA_1c_ levels higher than 48 mmol/mol (6.5%) at any time-point. For this study, additional exclusion criteria were that cases and controls had no known disease or used medication affecting steroid hormone homeostasis (for example glucocorticoid medication), however none of the participants reported this, resulting in a final sample of 116 cases (60 women and 56 men) and 138 controls (75 women and 63 men).

### Analysis of androgens

Serum samples from each survey, stored at − 70 °C, were thawed and analysed for three classic (A4, DHEAS, testosterone) and four 11-oxygenated androgens (11-hydroxytestosterone, 11OHT; 11-hydroxyandrostenedione, 11OHA4; 11-ketoandrostenedione, 11KA4; 11-ketotestosterone, 11KT) in April of 2021. The in-house laboratory method used for androgen analysis is currently an experimental method and is described in detail in the supplementary file (Table S1). Briefly, analyses were performed at the Arctic University of Norway, by liquid–liquid extraction (Tecan Fluent, Männedorf, Switzerland) and liquid chromatography (Waters Acquity™ *I*-class, Waters, Milford, Massachusettes, USA) interfaced with tandem mass spectrometry (Waters Xevo TQ-XS, Waters, Manchester, UK). MassChrom® Steroid panels (Chromsystems Instruments & Chemicals GmbH, München, Germany) and in-house spiked quality controls were included with each run for classic and 11-oxygenated androgens, respectively. All the standards and quality controls were within the acceptance limits of ± 15% from target value.

### Statistical analysis

Study sample characteristics are reported as means with standard deviations and/or frequencies with percentages, while androgen concentrations are reported as medians with interquartile ranges (IQRs). The distribution of androgen concentrations was assessed visually with histograms and tested for normality with the Shapiro–Wilks test, which indicated that none of the androgens were normally distributed. Differences in the characteristics of cases and controls were compared at each time-point using independent two-sample t-tests or Pearson’s χ2 test, while differences in the androgen concentrations were compared at each time-point using nonparametric Mann–Whitney U-test. All analyses were stratified by sex.

Following a review of the literature [21–26], directed acyclic graphs were constructed to identify potential confounding variables between androgens and T2DM (Figs. S2 and S3) Potential confounders identified for men were age, body mass index (BMI), and physical activity. The same confounders were identified for women, but also included age at menarche (years), parity (as a proxy for number of pregnancies), menstrual status (no; yes; uncertain/irregular), use of oral/intrauterine contraceptives (no; yes/previously), and use of oestrogens as hormone replacement therapy (no; yes; previously).

Generalised estimating equations, with log-link and gamma distribution to account for non-normality of the response variable, were used to assess androgen concentrations at each time-point and their time-trends. The concentrations were used as continuous dependent variables; T2DM status, the identified confounders, and an indicator variable for time with a two-way interaction term with T2DM, were used as independent variables. Unstructured correlation was used to address within-group correlation for the repeated measurements.

The association between androgen concentrations (per 1-IQR increment) and T2DM was assessed at each time-point by estimating odds ratios (ORs) using logistic regression models. Crude models included the respective androgen concentrations as continuous, independent variable, and T2DM status as the dependent variable. Adjusted models were identical to crude models but adjusted for the identified confounders.

To determine the potential for androgens to improve the prediction of T2DM, we constructed 3 types of logistic regression models: (1) A model including established risk factors alone (ERF model), (2) One that included established risk factors plus each investigated androgen respectively and (3) One with a combination of androgens (showed to be significant at any time-point in previous crude logistic regression models), chosen by backwards selection, in addition to established risk factors, with significantly improved model fit (by likelihood-ratio test) as criteria for inclusion of the androgens. ORs for androgen concentrations were evaluated by 1-IQR increments, and the ERF model included: Age (continuous), BMI (continuous), physical activity (active: ≥ 3 h/week of light activity and/or ≥ 1 h hard exercise/week and sedentary: < 3 h/week of activity that provoked transpiration or no activity), elevated blood pressure (systolic blood pressure ≥ 130, diastolic blood pressure ≥ 85, and/or use of blood pressure medication, yes/no), and family history of T2DM (siblings and/or parents with T2DM, yes/no) [27]. Models 2 and 3 were then compared to model 1 to identify improvements in predictive ability, assessed by area under the receiver operating characteristics (AROC), and interpreted as follows: an AROC of 0.50 indicates no discrimination, 0.50–0.70 poor discrimination, 0.70–0.80 acceptable discrimination, 0.80–0.90 excellent discrimination, and ≥ 0.90 outstanding discrimination [28].

Statistical analyses were performed in STATA (v 17.0, StataCorp LLC, 4905 Lakeway Drive, College Station, Texas, USA). Significance level was set at 5% with two-sided *p* values.

## Results

### Study sample characteristics

Detailed study sample characteristics that were reported in our previous study [20] and additional details about the specific covariates in the present study are included in the supplemental material (Table S2). For both men and women, cases and controls were similar in age, while cases had a significantly higher BMI (3.4–4.9 kg/m^2^ higher for women and 2.9–3.3 kg/m^2^ for men) at all time-points. Cases and controls had similar physical activity levels at all time-points, except at T2, when cases were less active than controls. For women, a higher percentage of cases than controls had elevated blood pressure and a family history of T2DM, while there were no significant differences in men. There were no significant differences in age at menarche, parity, menstrual status, or use of oral/intrauterine contraceptives in women.

### Androgen concentrations

**Fig. 1:**
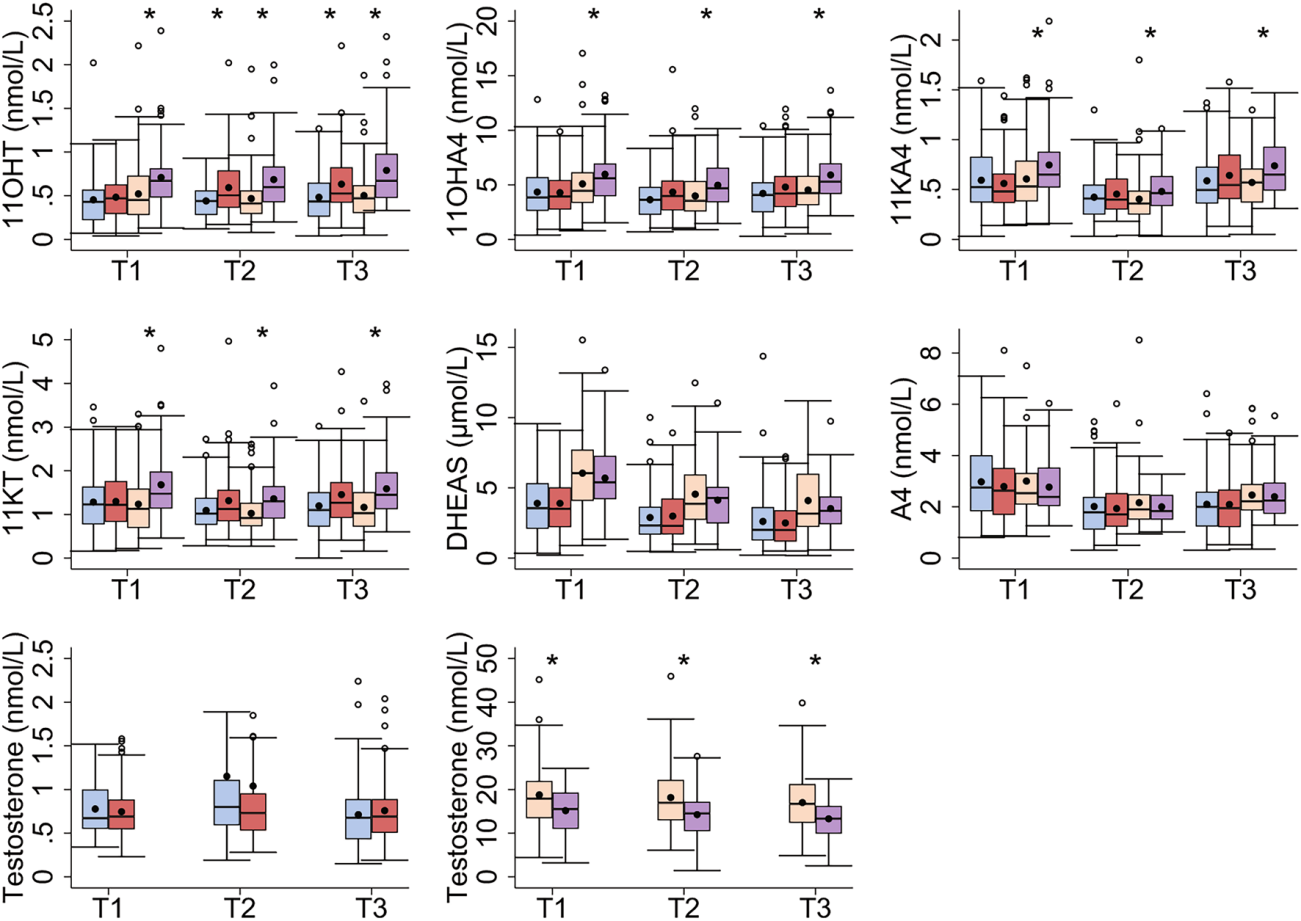
Androgen concentrations across all time-points in women (cases in red and controls in blue) and men (cases in purple and controls in orange). The Tromsø Study 1986–2001. Sample numbers at each time point: women: 60 cases and 75 controls; men: 56 cases and 63 controls. 11KA4, 11-ketoandrostenedione; 11KT, 11-ketotestosterone; 11OHT, 11-hydroxytestosterone; 11OHA4, 11-hydroxyandrostenedione; A4, androstenedione; DHEAS, dehydroepiandrosterone. T1, Tromsø3 (1986/87); T2, Tromsø4 (1994/95); T3, Tromsø5 (2001) *p < 0.05

For women, concentrations of 11OHT at T2 and T3 were significantly higher in cases than in controls; whereas for men, concentrations of 11OHT, 11OHA4, 11KA4, and 11KT were higher, and testosterone was lower at all time-points in cases than in controls (Fig. 1 and Table S3). After adjusting for confounders, no significant differences remained between women cases and controls; whereas for men, the differences between cases and controls remained significant for 11OHT (T2, T3), 11OHA4 (T3), 11KA4 (T1, T3), and 11KT (T1–T3) (Table S4). No significant differences between cases and controls were found for A4 and DHEAS.

### Longitudinal changes in androgen concentrations

**Fig. 2:**
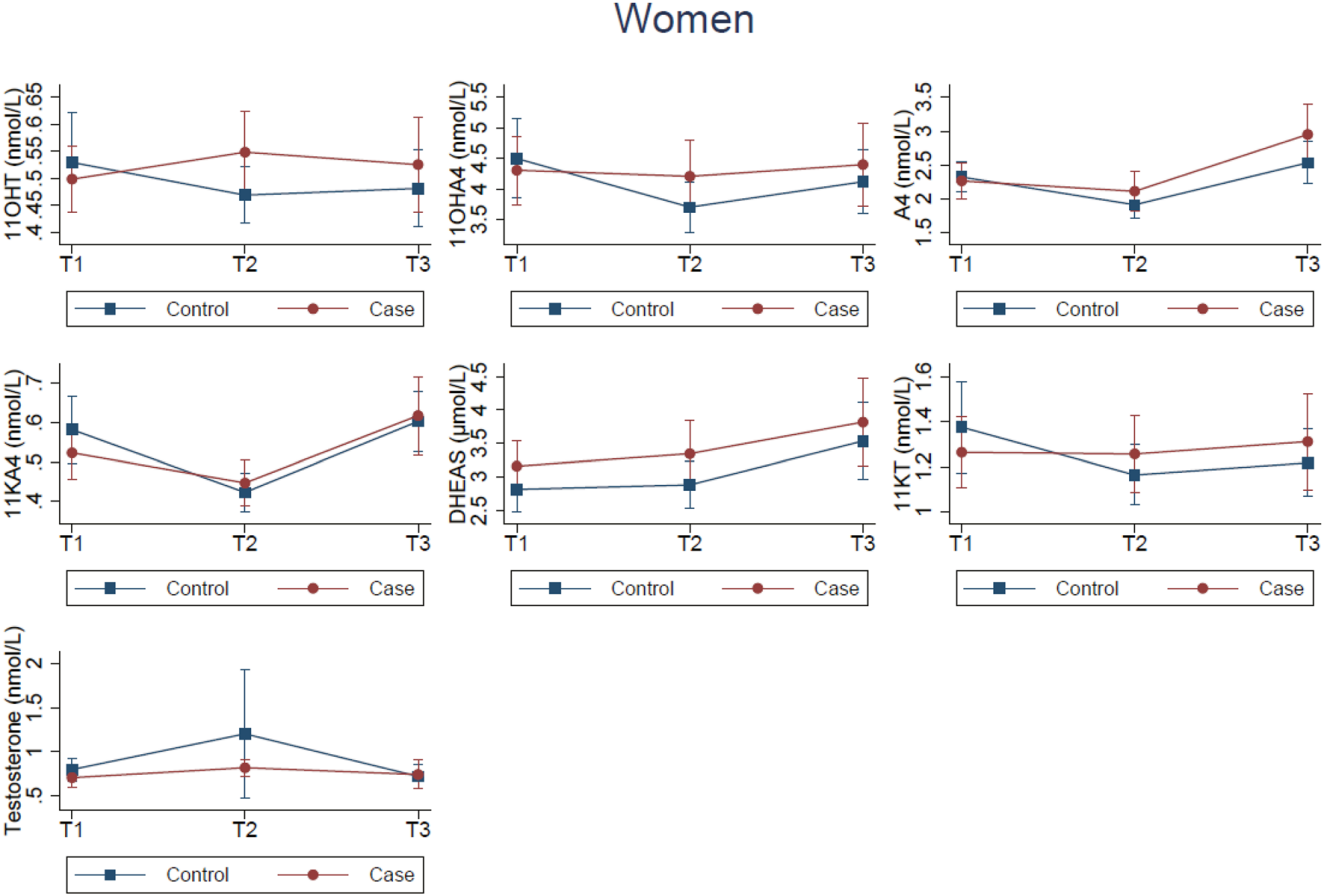
Estimated mean androgen concentrations across T1, T2, and T3 in women (60 cases and 75 controls at each time-point). The Tromsø Study 1986–2001. The estimated means in cases (red, circle) and controls (blue, square) are adjusted for age, body mass index, physical activity, age at menarche, parity, menstrual status, use of oral/intrauterine contraceptives, and use of hormone replacement therapy. T1, Tromsø3 (1986/87); T2, Tromsø4 (1994/95); T3, Tromsø5 (2001)

**Fig. 3:**
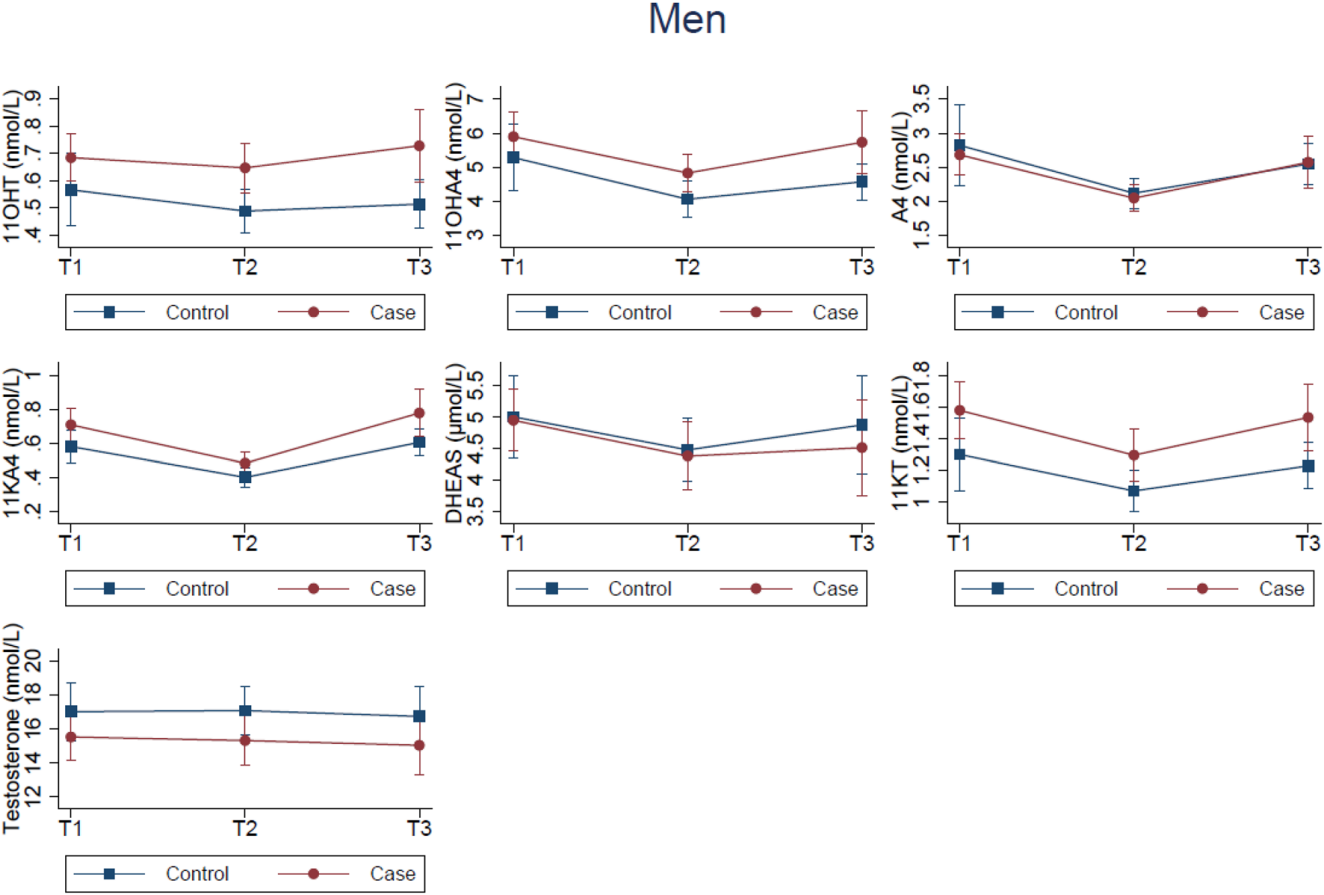
Estimated mean androgen concentrations across T1, T2, and T3 in men (56 cases and 63 controls at each time-point). The Tromsø Study 1986–2001. The estimated means for cases (red, circle) and controls (blue, square) are adjusted for age, body mass index, and physical activity. T1, Tromsø3 (1986/87); T2, Tromsø4 (1994/95); T3, Tromsø5 (2001)

For both men and women, no significant differences in changes in androgen concentrations over time were observed in cases compared to controls after adjusting for confounders (Figs. 2, 3 and Table S4).

### Associations between androgen concentrations and T2DM

**Fig. 4:**
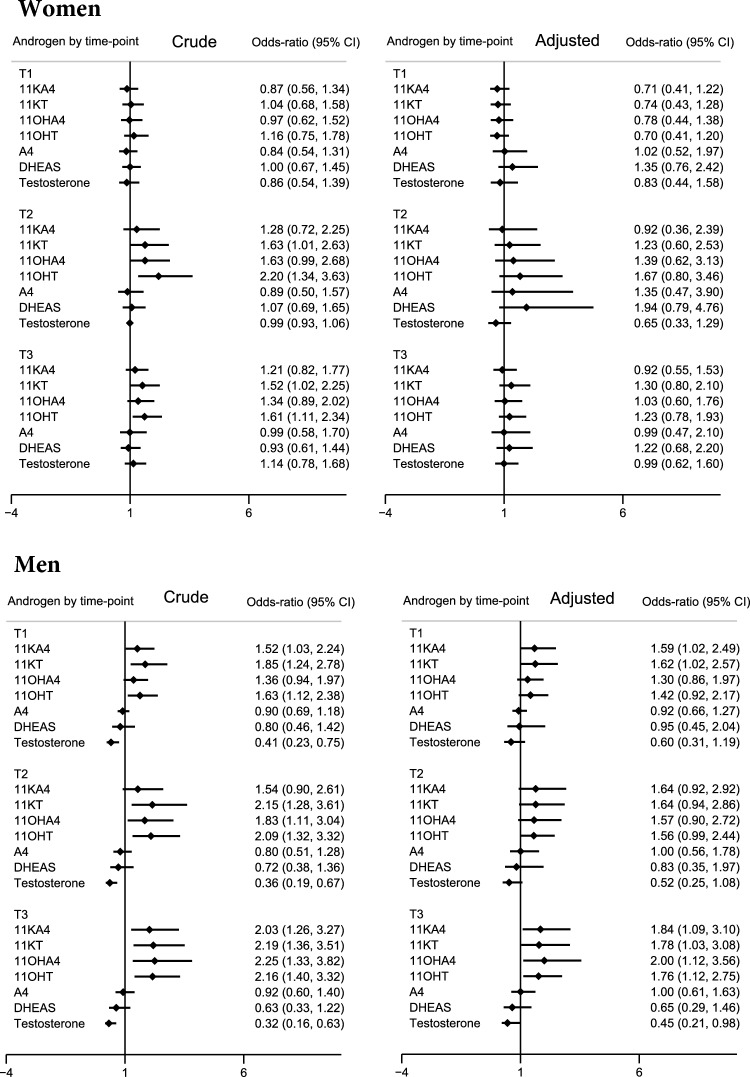
Forest plots illustrating crude and adjusted OR of type 2 diabetes for each androgen by time-point and sex. The Tromsø Study 1986–2001. Adjusted models included age, body mass index, and physical activity for both sexes. Models for women were further adjusted for age at menarche, parity, menstrual status, and use of oral/intrauterine contraceptives. OR for all androgens are estimated per 1-IQR increment. Sample numbers at each time point: women: 60 cases and 75 controls; men: 56 cases and 63 controls. 11KA4, 11-ketoandrostenedione; 11KT, 11-ketotestosterone; 11OHT, 11-hydroxytestosterone; 11OHA4, 11-hydroxyandrostenedione; A4, androstenedione; CI: confidence interval; DHEAS, dehydroepiandrosterone; IQR, interquartile range; T1, Tromsø3 (1986/87); T2, Tromsø4 (1994/95); T3, Tromsø5 (2001)

No significant associations were found between androgen concentrations and T2DM in the adjusted models for women (Fig. 4). For men, each 1-IQR increment in 11KA4 (T1, T3), 11KT (T1, T3), 11OHT (T3), and 11OHA4 (T3) significantly increased the OR of T2DM, while each 1-IQR increment in testosterone (T3) decreased these OR (Fig. 4 and Table S5).

### Potential of androgens to improve the prediction of T2DM

For men, the addition of 11KA4 or 11KT to the ERF model significantly improved the discrimination between cases and controls at T1 (79% vs 78%) (Table 1). Similar results were observed at T2 for the addition of 11OHT (80% discrimination vs 76%). At T3, the addition of 11OHT, 11OHA4, 11KA4, 11KT, or testosterone to the ERF model improved the discrimination between cases and controls (79–82% vs 78%).

**Table 1:** Ability of models including established risk factors alone, and established risk factors in addition to androgens, to predict T2DM across pre-diagnostic time-points. The Tromsø Study 1986–2001

	T1	T2	T3
Sex	AROC	AROC	AROC
Established risk factors^a^			
Women	0.77	0.81	0.80
Men	0.78	0.76	0.78
11-hydroxytestosterone^b^ (nmol/l)			
Women	0.77	0.82	0.80
Men	0.79	0.80*	0.82*
11-hydroxyandrostenedione^b^ (nmol/l)			
Women	–	–	–
Men	0.78	0.79	0.81*
11-ketoandrostenedione^b^ (nmol/l)			
Women	–	–	–
Men	0.79*	0.78	0.81*
11-ketotestosterone^b^ (nmol/l)			
Women	0.77	0.81	0.80
Men	0.79*	0.78	0.81*
Testosterone^b^ (nmol/l)			
Women	–	–	–
Men	0.78	0.78	0.79*
Combined model^c^			
Women	–	–	–
Men	–	–	0.85*

*AROC* Area Under the Receiver Operating Characteristics; *T*1 Tromsø3 (1986/87); *T*2 Tromsø4 (1994/95); *T*3 Tromsø5 (2001)

^*^Significant improvement in model fit by likelihood-ratio test between confounders and respective androgen plus confounders

^a^Established risk factors include age (continuous), body mass index (continuous), physical activity (active: ≥ 3 h/week of light activity and/or ≥ 1 h hard exercise/week or sedentary: < 3 h/week of activity that provoked transpiration or no activity), elevated blood pressure (systolic blood pressure ≥ 130, diastolic blood pressure ≥ 85, and/or if the subject was taking blood pressure medication, yes/no), and family history of type 2 diabetes (siblings and/or parents with type 2 diabetes, yes/no)

^b^Androgen included with the established risk factors

^c^Combined model for men at T3: established risk factors, 11OHT, and testosterone

Finally, when a combination of 11OHT and testosterone at T3 was added to the ERF model, a significant improvement over the models already presented was observed, showing 85% discrimination. At all other time-points, for both men and women, no androgen combinations improved discrimination or model fit more than those already presented.

## Discussion

To the best of our knowledge, this is the first study to address the relationship between pre-diagnostic measures of classic and 11-oxygenated androgens and T2DM with repeated measurements over a 15-years period. In men, after adjusting for confounders, cases had higher concentrations of 11-oxygentated androgens compared to controls throughout the study period and were positively associated with T2DM at T3 before its diagnosis in cases, whereas testosterone concentration was negatively associated with T2DM at T3. For women, after adjusting for confounders, there were no significant differences in androgen concentrations between cases and controls. In men, the addition of 11KA4, 11KT, 11OHT, 11OHA4, and testosterone, respectively, to the ERF model significantly improved discrimination between cases and controls, with acceptable to excellent discrimination (AROC 0.79 − 0.82) compared to acceptable discrimination (AROC 0.76 − 0.78) for the ERF model across all time-points. The strongest gain in discrimination was achieved by adding both 11OHT and testosterone to the ERF model at T3, with excellent discrimination (AROC 0.85) between cases and controls.

We observed that, in men, cases had significantly lower testosterone levels at all time-points compared to controls, and at T3 was significantly associated with T2DM after adjusting for confounders. This agrees with previous studies and meta-analysis, which found that decreased testosterone levels were associated with T2DM [12, 29]. No association was found between testosterone and T2DM for women, which also agrees with a previous meta-analysis [30]. In contrast to our results, some have observed that higher testosterone levels in women are associated with T2DM [12, 13]. Conversely, a study by O'Reilly et al. [12] with a median follow-up of 3.2–3.3 years found that lower levels of testosterone significantly increased the risk of T2DM for men (n: 70,541, mean age: 51.6 years) and that higher levels of testosterone increased the risk of T2DM for women (n: 81889, mean age: 33.2). Further, for women, they found that testosterone levels above 1.5 nmol/l increased the risk significantly. In the present study, the highest median testosterone concentration was 0.80 nmol/l, and few women had testosterone concentrations above 1.5 nmol/l, which may explain the discrepancy between these two studies. With 10 years of follow-up, Ding et al. [13] found a significant increase in the risk of T2DM in women with testosterone levels above 1.15 nmol/l (359 T2DM cases vs 359 controls, mean age: 60.3 years), which further indicates that testosterone levels higher than those seen in the present study are associated with T2DM.

Studies that have investigated the associations between 11-oxygenated androgens and T2DM in prospective cases are non-existent. In a study by Davio et al. [31], the aim was to compare 11-oxygenated androgen concentrations across adulthood in men and women in a general population, and they observed no associations with hyperglycaemia or T2DM in men and women. These findings agree with the non-significant associations we found in women, but not with our results in men. However, Davio et al. had a cross-sectional design that included prevalent T2DM cases (68 cases, 455 controls), whereas we studied T2DM cases before diagnosis. Some studies have examined the correlation between 11-oxygenated androgens and insulin, insulin resistance, and/or insulin sensitivity, however none of them included men. O'Reilly et al. [32] found that concentrations of 11OHA4 and 11KA4 were positively correlated with insulin and insulin resistance among healthy women and those with PCOS, and Walzer et al. [33] observed that, in women with lipodystrophy, 11OHA4, 11KA4, and 11KT, but not 11OHT, were associated with increased insulin signalling due to hyperinsulinemia as a response to insulin resistance. In contrast, Tosi et al. [6] found a positive correlation between insulin sensitivity and 11OHT and 11KT among women with PCOS. As disrupted insulin homeostasis is the major metabolic abnormality in T2DM, these observed correlations could be assumed to exist between 11-oxygenated androgens and T2DM.

In men, the addition of 11-oxygenated androgens and testosterone to the ERF model improved the prediction of T2DM (as early as 15 years before diagnosis for 11KA4 and 11KT). This suggests that elevated 11-oxygenated androgen concentrations and decreased testosterone concentrations might serve as biomarkers to identify individuals at high risk of T2DM when considered along with established risk factors. A study by Atlantis et al. [34] (n = 1655, median follow-up 4.95 years) compared a risk model consisting of combined variables from several risk models, with a risk model that additionally included testosterone and observed an increase in AROC from 0.82 to 0.83. They concluded that the discrimination was not significantly improved, but as testosterone remained significant, they presumed that its addition is valuable for identifying high-risk individuals.

In the present study, there were no significant differences in changes in androgen concentrations over time between cases and controls. This means that cases in men had higher concentrations compared to controls at T1, but the differences remained constant over the 15-year study period. These similar changes might be explained by the physiological properties of hormones. Indeed between-individual variations are generally greater than within-individual variations, and hormone homeostasis is tightly regulated within each individual by feedback mechanisms, thus a large difference in changes over time between cases and controls would not be expected [4]. The similar changes in androgens over time in cases and controls could indicate that androgen homeostasis was not disturbed during the study period. We do not know if the androgen concentrations were similar in cases and controls at an earlier time-point, and if so, when the difference in concentrations developed or what caused it. Hence, we cannot conclude whether the difference in androgen concentrations affected T2DM progression, or if it was a consequence of processes related to disease development.

Among men, cases had higher concentrations of 11-oxygenated androgens and lower concentrations of testosterone compared to controls, and these concentrations were positively and inversely associated with the development of T2DM, respectively. The opposite association with T2DM for 11-oxygenated androgens and testosterone may be explained by their different metabolic pathways, where the biosynthesis of 11-oxygenated androgens differs from that of testosterone. For example, obesity in men reduces the serum testosterone concentrations through increased storage in adipose tissue, an increased conversion of testosterone to oestrogens in adipose tissue through increased aromatase expression, and through reduced luteinising hormone stimulation of testosterone production [35, 36]. On the other hand, the production of 11-oxygenated androgens increases in obesity as their production are stimulated by aldo–keto reductase 1C3 (AKR1C3) which is expressed in adipose tissue [37, 38]. However, our result cannot confirm if these androgen concentrations are direct- or indirect risk factors for T2DM as they are interrelated to obesity which is a major risk factor for T2DM. Still, AKR1C3 expression has also been observed to be induced by insulin and insulin resistance, and it has been proposed that insulin may upregulate androgen biosynthesis, including 11-oxygenated androgens [39–42]. Further, it has been proposed that androgens activated by adipose tissue mediated by AKR1C3 also increased lipid synthesis that provokes lipotoxicity, which in turn is involved in insulin resistance progression [39]. Taken together, it is conceivable that 11-oxygenated androgens may be directly or indirectly associated with T2DM through the associations seen with BMI and insulin [43].

The concentrations of androgens reported in this study are comparable to concentrations reported in healthy subjects in several other studies [4, 31–33, 44, 45]. Even though we did not find any clear associations between androgens and T2DM in women in our study, associations have been observed in studies of women with PCOS, who have an excess of androgens, insulin sensitivity/resistance, and increased risk of developing T2DM [31, 32, 38, 44, 45]. The study by O'Reilly et al. [32], who observed significant relationships between insulin resistance and 11-oxygenated androgens, also observed that women with PCOS had concentrations between 1.34–5.0 times higher than controls. Androgen concentrations among women with T2DM in the present study were 0.9–1.2 times higher than in controls, which implies that androgen concentrations in women must be higher to detect significant associations and to be considered risk factors.

A major strength of this study is its nested case–control study design, with three repeated measurements available up to 15 years before T2DM diagnosis, and the wide array of data collection. T2DM diagnosis was determined by local diabetes registries and confirmed by medical records and HbA_1c_ results. Androgen measurements were based on analyses of serum samples by LC–MS/MS. Limitations include that external controls were only available for A4, DHEAS, and testosterone, but we did have in-house spiked controls for all androgens. Androgen concentrations are subject to circadian rhythm; therefore, it would have been favourable to adjust for the time of blood sampling which we did not have available. However, all participants followed the same enrolment procedures with similar order of events every day during the survey periods, thus cases and controls were likely treated equally, resulting in similar blood sampling times. We did have information on the date of participation for each individual which showed that blood collection was performed at similar months between cases and controls [20]. Stratifying by sex and time-point in the logistic regression models might have hampered the precision of our estimates and thus affected the interpretation of the results’ significance. The results are based on a northern Norwegian population and given the lack of studies and clinically relevant reference ranges for 11-oxygenated androgens, future studies are needed to re-evaluate the validity of our results.

## Conclusions

For men, prospective T2DM cases had consistently higher concentrations of 11-oxygenated androgens, and lower testosterone concentrations compared to controls. Further, several androgens improved the discrimination of cases and controls in prediction models, indicating that androgens may be potential biomarkers in T2DM risk assessment. Still, we cannot conclude if androgens affect T2DM progression, or whether concentrations are affected by other factors related to disease development.

### Supplementary Information

Below is the link to the electronic supplementary material.

Supplementary file1 (DOCX 270 KB)

## Data Availability

The dataset used in this study was procured from the Tromsø Study and is not publicly available. Access may be obtained by application to the Tromsø Study (https://uit.no/research/tromsostudy).
