# Supplementary material for: Longitudinal assessment of classic and 11-oxygenated androgen concentrations and their association with type 2 diabetes mellitus development: the Tromsø study
Source: Acta Diabetol. 2024 Mar 18;61(7):847–57. doi: 10.1007/s00592-024-02266-5 (PMC11182793; doi:10.1007/s00592-024-02266-5)
Supplement: Supplementary file 1 — Supplementary file1 (DOCX 270 KB) [file 592_2024_2266_MOESM1_ESM.docx]

**Supplemental material**

**Longitudinal assessment of classic and 11-oxygenated androgen concentrations and their association with Type 2 Diabetes Mellitus development – The Tromsø Study**

Giovanni Allaoui^ab^, Charlotta Rylander^c^, Ole-Martin Fuskevåg^ad^, Guri Grimnes^de^, Maria Averina^ad^, Tom Wilsgaard^c^, Vivian Berg^ab*^

^a^Department of Laboratory Medicine, Division of Diagnostic Services, University Hospital of North-Norway, NO-9038 Tromsø, Norway

^b^Department of Medical Biology, Faculty of Health Sciences, UiT-The Arctic University of Norway, NO-9037 Tromsø, Norway

^c^Department of Community Medicine, Faculty of Health Sciences, UIT-The Arctic University of Norway, NO-9037 Tromsø, Norway

^d^Department of Clinical Medicine, Faculty of Health Sciences, UIT-The Arctic University of Norway, NO-9037 Tromsø, Norway

^e^Division of Medicine, University Hospital of North-Norway, NO-9038 Tromsø, Norway

***Correspondence:**

Vivian Berg, Department of Medical Biology, Faculty of Health Sciences, UiT-The Arctic University of Norway, NO-9037 Tromsø, Norway

Phone: +47 77 64 46 77

Email: vivian.berg@uit.no

**Method description for liquid chromatography – tandem mass spectrometry setup (LC-MS/MS)**

*Chemicals*

LC-grade tert-butyl methyl ether (TBME) and LC-MS/MS-grade methanol was purchased from Merck KGaA (Dramstadt, Germany), zinc sulphate heptahydrate (ZnSO_4_*7H_2_O) from Acros Organics, LC-MS/MS-grade formic acid from Thermo Scientific, and Milli-Q grade water was produced by a Millipore system.

The analytes 17a-hydroxyprogesterone (**1**), testosterone (**2**), progesterone (**3**), 21-deoxycortisol (**4**), 11-deoxycortisol (**5**), corticosterone (**6**), and cortisol (**7**) were purchased from Cerilliant Corporation (Round Rock, Texas, USA); androstenedione (**8**) from LGC Germany; 4-androsten-11B-17B-diol-3one (11-hydroxytestosterone) (**9**), 4-androsten-11B-OL3-17-dione (11-hydroxyandrostenedione) (**10**), 4-androsten-3-11-17-trione (11-ketoandrostenedione (**11**), 4-androsten-17B-OL3-11B-dione (11-ketotestosterone) (**12**), from Steraloids Inc. (Newport, Rhode Island, USA); and dehydroepiandrosterone sulphate (**13**) from Steraloids Inc. (Newport, Rhode Island, USA).

The isotope labelled analytes 17a-hydroxyprogesterone-d8 (**1***), testosterone-d3 (**2***), progesterone-d9 (**3***), 21-deoxycortisol-d8 (**4***), 11-deoxycortisol-d5 (**5***), corticosterone-d8 (**6***), and dehydroepiandrosterone sulphate-d5 (**13***) were purchased from Cerilliant Corporation (Round Rock, Texas, USA); cortisol-d4 (**7***) from IsoSciences (Ambler, Pennsylvania, USA); androstenedione-d3 (**8***) from TRC Canada (Toronto, Ontario, Canada); 4-androsten-11B-17B-diol-3one-d4 (11-hydroxytestosterone**-**d4) (**9***), 4-androsten-11B-OL3-17-dione-d4 (11-hydroxyandrostenedione-d4) (**10***), and 4-androsten-3-11-17-trione-d10 (11-ketoandrostenedione-d10) (**11***) from Cambridge Isotope Laboratories (Andover, Massachusetts, USA); and 4-androsten-17B-OL3-11B-dione-d3 (11-ketotestosterone-d3 (**12***) from Cayman Chemical Group (Ann Arbor, Michigan, USA).

For quality control (QC) of analytes **1-8** and **13**, the CE-IVD MassChrom® Steroids panel 1 and 2 with three levels each were purchased from Chromsystems Instruments & Chemicals (München, Germany). For the 11-oxygenated androgens, concentrations for low controls were spiked to 2 nM, and for high controls were spiked to 20 nM.

*Sample preparation*

A seven-point calibration curve ranging from 0.13 to 130 nM for analytes **1, 2, 3, 5, 6,** and **8**; from 0.013 to 13 nM for analyte **4**; from 2.08 to 2080 nM for analyte **7;** from 0.025 to 25 nM for analytes **9, 10, 11,** and **12**; and from 26 to 26000 nM for analyte **13** was prepared in methanol:water (1:1). The isotope labelled analytes were used as internal standards (IS), where 2*,8*-12*; 7*; and 13* were mixed to a concentration of 3, 30, and 100 nmol/l, respectively, in ultrapure H_2_O.

Extraction was performed on a Tecan Fluent 780 liquid handler. 70 µl of sample, calibration standard, and QC samples were transferred to a 96-well plate (Sarstedt) whereafter 60 µl IS-mix was added and 110 µl 0.1 M ZnSO_4_:methanol (1:1) for protein precipitation. After shaking at 1500 RPM for 2 minutes, 500 µl TBME was added, and the samples were shaken at 1450 RPM for 3 minutes for liquid-liquid extraction of steroids. The plates were centrifuged for 4 minutes at 1600 RPM (Hettich Rotina 320R) and the upper organic phase was transferred to a 1 ml, 96-well sample collection plate (Waters). The solvent was evaporated under a stream of nitrogen while kept on 40°C. Finally, the samples were reconstituted in 60 µl of 70% methanol with 0.1% formic acid.

*LC-MS/MS analysis*

Separation was achieved with a Cortecs T3, 120Å, 1.6 µm 2.1 x 100 mm (Waters) column, maintained at 50°C with a flow rate of 0.3 mL/min. A linear gradient system composed of 0.1% formic acid and 5 mM ammonium acetate in water, and 0.1% formic acid and 5 mM ammonium acetate in methanol:acetonitrile (1:1) was used, starting from 40% (v/v) methanol, and increasing to 70% in 8 min, maintaining at 95% for 0.5 min before returning to the starting conditions. The autosampler temperature was 6°C and the sample injection volume was 4 μl. The injector was a flow-through-needle (FTN), the needle wash and purge solvent were 90% methanol, and the needle was washed for 6 seconds after injection. Mass spectral data was acquired on a Waters Xevo TQ-XS mass spectrometer (Waters, Manchester, UK) in ESI positive and negative mode with the following conditions: capillary voltage: 1 kV, desolvation gas temperature: 550°C, source temperature: 150°C, desolvation gas flow: 1000 L/hr, cone gas flow: 150 l/hr, nebuliser pressure: 7 Bar. Table S1 shows the retention time (RT), multiple reaction monitoring (MRM) transitions, cone voltage and collision energy used for the different analytes. Data were acquired and analysed using MassLynx version 4.2.

*Validation*

No formal validation of the method has been conducted, as it was an experimental method. However, in initial assessments, by using the mentioned calibration curve and controls, it was observed a linearity of R^2^>0.99, an intraday precision of <7% and an intermediate precision of <10%. The LLOQ for each analyte was the lowest level of the calibration curve.

Table S1. Retention time (RT), multiple reaction monitoring (MRM) transitions, cone voltage, and collision energy used for the different analytes.

| **nr** | **Analyte** | **RT (min)** | **Quantifier MRM transitions (m/z)** | **Qualifier MRM transitions (m/z)** | **Cone (V)** | **Collision (eV)** |
| --- | --- | --- | --- | --- | --- | --- |
| 1  1* | 17a-OH-progesterone  17a-OH-progesterone-d8 | 6.74  6.66 | 331.1>97  339.1>100 | 331.1>109 | 40 | 23/23  23 |
| 2  2* | Testosterone  Testosterone-d3 | 6.31  6.26 | 289.1>97  292.1>97 | 289.1>109  292.1>109 | 40 | 24/24  24/24 |
| 3  3* | Progesterone  Progesterone-d9 | 8.37  8.27 | 315.1>109  324.1>113 | 315.1>97  324.1>100 | 40 | 23/23  23/23 |
| 4  4* | 21-Deoxycortisol  21-Deoxycortisol-d8 | 4.77  4.69 | 347.1>121  355.4>113 | 347.1>311  355.4>319 | 20 | 21/21  18/21 |
| 5  5* | 11-Deoxycortisol  11-Deoxycortisol-d5 | 4.99  4.93 | 347.1>109  352.1>113 | 347.1>97  352.1>100 | 40 | 23/24  24/24 |
| 6  6* | Corticosterone  Corticosterone-d8 | 4.77  4.69 | 347.2>329  355.2>337 | 347.2>121  355.2>125 | 20 | 18/20  21/21 |
| 7  7* | Cortisol  Cortisol-d4 | 3.63  3.60 | 363.1>121  367.1>121 | 363.1>327  367.1>331 | 40 | 25/14  25/14 |
| 8  8* | Androstenedione  Androstenedione-d3 | 5.78  5.73 | 287.1>109  290.1>109 | 287.1>97  290.1>100 | 40 | 23/23  23/23 |
| 9  9* | 4-Androsten-11B-17B-diol-3one  4-Androsten-11B-17B-diol-3one-d4 | 4.60  4.56 | 305.2>121  309.2>121 | 305.2>287  309.2>291 | 40 | 17/17  17/17 |
| 10  10* | 4-Androsten-11B-OL3-17-dione  4-Androsten-11B-OL3-17-dione-d4 | 4.22  4.18 | 303.2>267  307.2>270 | 303.2>285  307.2>289 | 40 | 18/18  21/21 |
| 11  11* | 4-Androsten-3-11-17-trione  4-Androsten-3-11-17-trione-d10 | 3.46  3.41 | 301.2>257  311.2>125 | 301.2>121  311.2>265 | 40 | 23/23  23/23 |
| 12  12* | 4-Androsten-17B-OL3-11B-dione  4-Androsten-17B-OL3-11B-dione-d3 | 3.89  3.86 | 303.2>121  306.2>121 | 303.2>259  306.2>262 | 40 | 23/23  23/23 |
| 13  13* | Dehydroepiandrosterone sulphate Dehydroepiandrosterone sulphate-d5 | 2.10 | 367.1>97  372.1>98 |  |  |  |


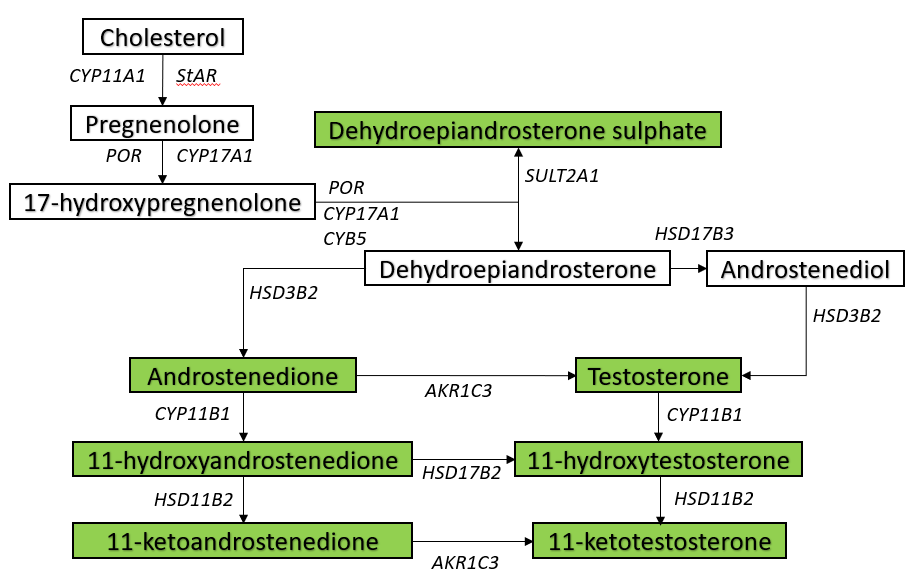
**Fig. S1.** Pathway schematic of androgen biosynthesis with corresponding genes for the key enzymes in the steroidogenic pathway. AKR1C3, Aldo-keto reductase family 1 member C3; CYB5, cytochrome b_5_; CYP11A1, cytochrome P450 family 11 subfamily A member 1; CYP11B1, cytochrome P450 family 11 subfamily B member 1; CYP17A1, cytochrome P450 family 17 subfamily A member 1; HSD3B2, 3 beta-hydroxysteroid dehydrogenase type 2; HSD11B2, 11 beta-hydroxysteroid dehydrogenase type 2 HSD17B2, 17 beta-hydroxysteroid dehydrogenase type 2; HSD17B3, 17 beta-hydroxysteroid dehydrogenase type 3; POR, cytochrome P450 oxidoreductase; StAR, steroidogenic acute regulatory protein; SULT2A1, sulfotransferase family 2A member 1.


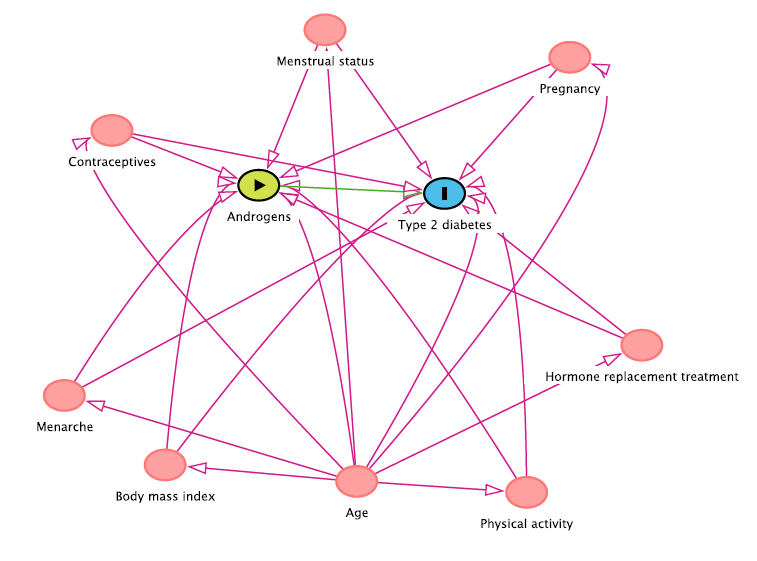
**Fig. S2.** Directed acyclic graph of assumptions of associations between androgens and type 2 diabetes for women, illustrating potential confounders. Red circles illustrate confounders, the green circle illustrates the exposure, and the blue circle is the outcome.


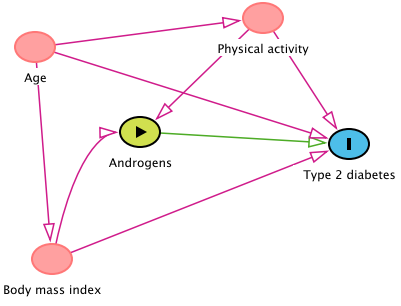


**Fig. S3.** Directed acyclic graph of assumptions of associations between androgens and type 2 diabetes for men, illustrating potential confounders. Red circles illustrate confounders, the green circle illustrates the exposure, and the blue circle is the outcome.

Table S2. Study sample characteristics by time-point. The Tromsø Study 1986– 2001.

|  |  |  | **T1 1986/87** |  | **T2**  **1994/95** |  | **T3**  **2001** |  |
| --- | --- | --- | --- | --- | --- | --- | --- | --- |
|  |  |  | **Mean (SD)** | **ΔMean**  **case-control**  **(95% CI)** | **Mean (SD)** | **ΔMean**  **case-control**  **(95% CI)** | **Mean (SD)** | **ΔMean**  **case-control**  **(95% CI)** |
| Age (years) | Women | Case | 46.3 (6.36) | 2.50  (-0.19, 5.19) | 54.3 (6.36) | 2.50  (-0.19, 5.19) | 61.3 (6.36) | 2.50  (-0.19, 5.19) |
|  |  | Control | 43.8 (8.88) |  | 51.8 (8.88) |  | 58.8 (8.88) |  |
|  | Men | Case | 48.8 (8.66) | 2.09  (-1.49, 5.64) | 56.8 (8.66) | 2.09  (-1.49, 5.64) | 63.8 (8.66) | 2.09  (-1.49, 5.64) |
|  |  | Control | 46.7 (10.7) |  | 54.7 (10.7) |  | 61.7 (10.7) |  |
| BMI (kg/m^2^) | Women | Case | 27.1 (4.27) | 3.35  (1.97, 4.72) | 29.2 (4.91) | 4.21  (2.58, 5.83) | 31.2 (5.69) | 4.88  (3.12, 6.63) |
|  |  | Control | 23.7 (3.75) |  | 25.0 (4.61) |  | 26.4 (4.60) |  |
|  | Men | Case | 27.6 (3.49) | 2.92  (1.79, 4.05) | 28.7 (3.44) | 3.01  (1.84, 4.18) | 29.8 (3.52) | 3.28  (2.02, 4.55) |
|  |  | Control | 24.7 (2.72) |  | 25.7 (3.02) |  | 26.6 (3.44) |  |
| Menarche age (years) | Women | Case | 13.4 (1.26) | -0.02  (-0.46, 0.50) | 13.4 (1.26) | -0.02  (-0.46, 0.50) | 13.4 (1.26) | -0.02  (-0.46, 0.50) |
|  |  | Control | 13.3 (1.50) |  | 13.3 (1.50) |  | 13.3 (1.50) |  |
| Parity (n) | Women | Case | 2.79 (0.20) | 0.39  (-0.15, 0.93) | 2.93 (0.19) | 0.41  (-0.10, 0.92) | 2.95 (0.20) | 0.33  (-0.18, 0.85) |
|  |  | Control | 2.40 (0.18) |  | 2.55 (0.18) |  | 2.61 (0.17) |  |
|  |  |  | **n (%)** | ***p*-value** | **n (%)** | ***p*-value** | **n (%)** | ***p*-value** |
| Physical activity: Women | Active | Case | 48 (80.0) | 0.71 | 26 (43.3) | <0.01 | 44 (74.6) | 0.64 |
|  |  | Control | 58 (77.3) |  | 51 (68.0) |  | 57 (78.1) |  |
|  | Inactive | Case | 12 (20.0) |  | 34 (56.7) |  | 15 (25.4) |  |
|  |  | Control | 17 (22.7) |  | 24 (32.0) |  | 16 (21.9) |  |
| Physical activity: Men | Active | Case | 46 (82.1) | 0.96 | 39 (69.6) | 0.55 | 42 (77.8) | 0.62 |
|  |  | Control | 52 (82.5) |  | 47 (74.6) |  | 45 (73.8) |  |
|  | Inactive | Case | 10 (17.9) |  | 17 (30.4) |  | 12 (22.2) |  |
|  |  | Control | 11 (17.5) |  | 16 (25.4) |  | 16 (26.2) |  |
| Menstrual status: Women | Yes | Case | 35 (58.3) | 0.85 | 12 (20.0) | 0.08 | 5 (8.3) | 0.16 |
|  |  | Control | 42 (56.0) |  | 25 (33.3) |  | 7 (9.3) |  |
|  | No | Case | 21 (35.0) |  | 48 (80.0) |  | 54 (90.0) |  |
|  |  | Control | 26 (34.7) |  | 48 (64.0) |  | 61 (81.3) |  |
|  | Uncertain/  Irregular | Case | 4 (6.7) |  | 0 (0.0) |  | 1 (1.7) |  |
|  |  | Control | 7 (9.3) |  | 2 (2.7) |  | 7 (5.3) |  |
| Contraceptives: Women | No | Case | 25 (43.9) | 0.82 | 28 (56.0) | 0.52 | 39 (68.4) | 0.83 |
|  |  | Control | 31 (41.9) |  | 32 (50.0) |  | 50 (66.7) |  |
|  | Yes/previously | Case | 32 (56.1) |  | 28 (56.0) |  | 18 (31.6) |  |
|  |  | Control | 43 (58.1) |  | 32 (50.0) |  | 25 (33.3) |  |
| Hormone replacement treatment: Women | No | Case | 54 (96.4) | 0.80 | 41 (74.6) | 0.20 | 29 (50.0) | 0.78 |
|  |  | Control | 70 (97.2) |  | 42 (60.0) |  | 34 (45.3) |  |
|  | Yes | Case | 2 (3.6) |  | 11 (20.0) |  | 22 (37.9) |  |
|  |  | Control | 2 (2.8) |  | 24 (34.3) |  | 33 (44.0) |  |
|  | Previously | Case | -- |  | 3 (5.5) |  | 7 (12.1) |  |
|  |  | Control | -- |  | 4 (5.7) |  | 8 (10.7) |  |
| Elevated blood pressure: Women | No | Case | 22 (36.7) | <0.01 | 17 (28.3) | 0.02 | 10 (16.7) | <0.01 |
|  |  | Control | 50 (66.7) |  | 36 (48.0) |  | 32 (42.7) |  |
|  | Yes | Case | 38 (63.3) |  | 43 (71.7) |  | 50 (83.3) |  |
|  |  | Control | 25 (33.3) |  | 39 (52.0) |  | 43 (57.3) |  |
| Elevated blood pressure: Men | No | Case | 13 (23.2) | 0.22 | 11 (19.6) | 0.45 | 11 (19.6) | 0.73 |
|  |  | Control | 21 (33.3) |  | 16 (25.4) |  | 14 (22.2) |  |
|  | Yes | Case | 43 (76.8) |  | 45 (80.4) |  | 45 (80.4) |  |
|  |  | Control | 42 (66.7) |  | 47 (74.6) |  | 49 (77.8) |  |
| Family history of type 2 diabetes: Women | No | Case | 45 (75.0) | 0.03 | 38 (63.3) | <0.01 | 35 (58.3) | 0.01 |
|  |  | Control | 67 (89.3) |  | 65 (86.7) |  | 60 (80.0) |  |
|  | Yes | Case | 15 (25.0) |  | 22 (36.7) |  | 25 (41.7) |  |
|  |  | Control | 8 (10.7) |  | 10 (13.3) |  | 15 (20.0) |  |
| Family history of type 2 diabetes: Men | No | Case | 45 (80.4) | 0.59 | 42 (75.0) | 0.57 | 39 (69.6) | 0.42 |
|  |  | Control | 53 (84.1) |  | 50 (79.4) |  | 48 (76.2) |  |
|  | Yes | Case | 11 (19.6) |  | 14 (25.0) |  | 17 (30.4) |  |
|  |  | Control | 10 (15.9) |  | 13 (20.6) |  | 15 (23.8) |  |

Sample numbers at each time point: women: 60 cases and 75 controls; men: 56 cases and 63 controls. BMI, body mass index; CI, confidence interval; SD, standard deviation; T1, Tromsø3 (1986/87); T2, Tromsø4 (1994/95); T3, Tromsø5 (2001).

Table S3. Androgen concentrations at all time-points in men and women comparing cases and controls. Results presented as median with IQR. The Tromsø Study 1986–2001.

|  |  |  | **Pre-diagnostic time-points** | | | | | |
| --- | --- | --- | --- | --- | --- | --- | --- | --- |
|  |  |  | **T1**  **1986/87** | | **T2**  **1994/95** | | **T3**  **2001** | |
| **Hormone** |  |  | **Median (IQR)** | **p-value** | **Median (IQR)** | **p-value** | **Median (IQR)** | **p-value** |
| **11OHT (nmol/l)** | Women^a^  Men^b^ | Case  Control  Case  Control | 0.47 (0.34)  0.43 (0.35)  0.67 (0.34)  0.45 (0.45) | 0.13  <0.01 | 0.51 (0.43)  0.44 (0.28)  0.60 (0.41)  0.41 (0.27) | <0.01  <0.01 | 0.53 (0.41)  0.44 (0.39)  0.67 (0.51)  0.47 (0.32) | 0.01  <0.01 |
| **11OHA4 (nmol/l)** | Women  Men | Case  Control  Case  Control | 3.94 (2.71)  3.83 (3.08)  5.60 (3.00)  4.44 (2.80) | 0.98  0.04 | 3.98 (2.74)  3.63 (2.56)  4.68 (3,17)  3.53 (2.80) | 0.10  0.01 | 4.21 (2.84)  4.06 (2.76)  5.29 (2,81)  4.24 (2.69) | 0.22  <0.01 |
| **A4**  **(nmol/l)** | Women  Men | Case  Control  Case  Control | 2.63 (1.83)  2.75 (2.18)  2.39 (1.50)  2.53 (1.23) | 0.28  0.60 | 1.71 (1.31)  1.79 (1,28)  1.84 (0.97)  1.90 (0.99) | 0.96  0.79 | 1.95 (1,47)  2.00 (1.36)  2.25 (1.22)  2.22 (1.03) | 0.85  0.71 |
| **11KA4 (nmol/l)** | Women  Men | Case  Control  Case  Control | 0.48 (0.30)  0.53 (0,46)  0.65 (0.36)  0.53 (0,41) | 0.34  0.01 | 0.40 (0,31)  0.41 (0.30)  0.47 (0.30)  0.36 (0.24) | 0.39  0.02 | 0.55 (0.45)  0.50 (0.37)  0.65 (0.44)  0.57 (0.34) | 0.33  <0.01 |
| **DHEAS**  **(µmol/l)** | Women  Men | Case  Control  Case  Control | 3.52 (2.83)  3.55 (3.24)  5.40 (3.08)  6.05 (3.64) | 0.90  0.46 | 2.30 (2.55)  2.32 (2.00)  4.29 (2.61)  3.86 (3.24) | 0.77  0.53 | 2.00 (2.23)  2.02 (2.38)  3.37 (1.99)  3.18 (3.79) | 0.96  0.56 |
| **11KT (nmol/l)** | Women  Men | Case  Control  Case  Control | 1.22 (0.94)  1.23 (0,87)  1.48 (0.85)  1.13 (0.90) | 0.97  <0.01 | 1.13 (0.72)  1.02 (0.62)  1.30 (0.75)  0.92 (0.54) | 0.09  <0.01 | 1.27 (0.82)  1.10 (0.79)  1.45 (0.85)  1.03 (0.79) | 0.06  <0.01 |
| **Testosterone**  **(nmol/l)** | Women  Men | Case  Control  Case  Control | 0.69 (0.34)  0.67 (0.45)  15.5 (8.31)  17.9 (8.52) | 0.61  0.01 | 0.73 (0.43)  0.80 (0.52)  14.5 (6.71)  16.9 (9.28) | 0.28  <0.01 | 0.69 (0.39)  0.68 (0.46)  13.3 (6.30)  16.7 (8.91) | 0.58  <0.01 |

Sample numbers at each time point: women: 60 cases and 75 controls; men: 56 cases and 63 controls. 11KA4, 11-ketoandrostenedione; 11KT, 11-ketotestosterone; 11OHT, 11-hydroxytestosterone; 11OHA4, 11-hydroxyandrostenedione; A4, androstenedione; DHEAS, dehydroepiandrosterone sulphate; IQR, inter quartile range; T1, Tromsø3 (1986/87); T2, Tromsø4 (1994/95); T3, Tromsø5 (2001).

Table S4. Androgen concentrations at all time-points, adjusted for confounders^a^. Models were generalised estimating equations with log-link and gamma distribution. Androgen concentrations were the dependent variable with time-points and confounders as independent variables. T3 is set as the reference time-point. The Tromsø Study 1986–2001.

| **Biomarker** |  | **Men** | | | **Women** | | |
| --- | --- | --- | --- | --- | --- | --- | --- |
|  |  | **β-coefficient**  **(SE)** | **p-value** | **95% confidence interval** | **β-coefficient**  **(SE)** | **p-value** | **95% confidence interval** |
| **11OHT (nmol/l)** | Case  T1  T2  T3  Case#T1  Case#T2  Case#T3  Constant | 0.35 (0.12)  0.10 (0.14)  -0.05 (0.09)  *Reference*  -0.16 (0.12)  -0.07 (0.11)  *Reference*  -1.55 (0.55) | <0.01  0.49  0.59  --  0.17  0.55  --  0.01 | 0.12, 0.58  -0.18, 0.37  -0.24, 0.13  --  -0.39, 0.07  -0.29, 0.15  --  -2.63, -0.47 | 0.08 (0.10)  0.10 (0.12)  -0.01 (0.07)  *Reference*  -0.17 (0.11)  0.06 (0.09)  *Reference*  -1.95 (0.44) | 0.45  0.39  0.86  --  0.13  0.53  --  <0.01 | -0.13, 0.28  -0.13, 0.33  -0.16, 0.13  --  -0.39, 0.05  -0.12, 0.24  --  -2.81, -1.08 |
| *Adjusted cases compared to controls at single time-points* | T1  T2  T3 | 0.19 (0.12)  0.28 (0.12)  0.35 (0.12) | 0.11  0.02  <0.01 | -0.04, 0.42  0.05, 0.51  0.12, 0.58 | -0.09 (0.09)  0.14 (0.09)  0.08 (0.10) | 0.34  0.13  0.45 | -0.28, 0.10  -0.04, 0.32  -0.13, 0.28 |
| **11OHA4 (nmol/l)** | Case  T1  T2  T3  Case#T1  Case#T2  Case#T3  Constant | 0.23 (0.09)  0.15 (0.11)  -0.12 (0.06)  *Reference*  -0.12 (0.11)  -0.05 (0.09)  *Reference*  1.22 (0.46) | 0.02  0.17  0.06  --  0.27  0.56  --  0.01 | 0.04, 0.41  -0.06, 0.35  -0.24, 0.01  --  -0.33, 0.09  -0.23, 0.13  --  0.32, 2.12 | 0.03 (0.09)  0.05 (0.09)  -0.13 (0.09)  *Reference*  -0.10 (0.10)  0.07 (0.08)  *Reference*  0.82 (0.41) | 0.71  0.56  0.04  --  0.35  0.43  --  0.05 | -0.15, 0.22  -0.12, 0.23  -0.26, -0.004  --  -0.30, 0.11  -0.10, 0.23  --  0.02, 1.63 |
| *Adjusted cases compared to controls at single time-points* | T1  T2  T3 | 0.11 (0.10)  0.17 (0.09)  0.23 (0.09) | 0.27  0.06  0.02 | -0.08, 0.30  -0.004, 0.35  0.04, 0.41 | -0.05 (0.09)  0.10 (0.09)  0.03 (0.09) | 0.50  0.25  0.71 | -0.24, 0.12  -0.07, 0.27  -0.15, 0.22 |
| **A4**  **(nmol/l)** | Case  T1  T2  T3  Case#T1  Case#T2  Case#T3  Constant | 0.01 (0.08)  0.10 (0.12)  -0.18 (0.05)  *Reference*  -0.06 (0.10)  -0.05 (0.08)  *Reference*  1.53 (0.43) | 0.89  0.41  <0.01  --  0.52  0.54  --  <0.01 | -0.14, 0.16  -0.14, 0.35  -0.29, -0.08  --  -0.19, 0.13  -0.19, 0.10  --  0.68, 2.38 | 0.10 (0.09)  -0.15 (0.07)  -0.30 (0.05)  *Reference*  -0.12 (0.10)  -0.03 (0.08)  *Reference*  1.73 (0.31) | 0.28  0.04  <0.01  --  0.25  0.74  --  <0.01 | -0.08, 0.28  -0.30, -0.01  -0.41, -0.19  --  -0.33, 0.09  -0.19, 0.13  --  1.12, 2.35 |
| *Adjusted cases compared to controls at single time-points* | T1  T2  T3 | -0.05 (0.10)  -0.04 (0.07)  0.01 (0.08) | 0.62  0.62  0.89 | -0.25, 0.15  -0.17, 0.10  -0.14, 0.16 | -0.02 (0.07)  0.07 (0.08)  0.10 (0.09) | 0.80  0.39  0.28 | -0.17, 0.13  -0.09, 0.24  -0.08, 0.28 |
| **11KA4 (nmol/l)** | Case  T1  T2  T3  Case#T1  Case#T2  Case#T3  Constant | 0.25 (0.10)  -0.04 (0.10)  -0.41 (0.06)  *Reference*  -0.05 (0.11)  -0.06 (0.10)  *Reference*  -0.20 (0.53) | 0.01  0.68  <0.01  --  0.62  0.53  --  0.70 | 0.05, 0.45  -0.24, 0.16  -0.53, -0.29  --  -0.26, 0.15  -0.26, 0.13  --  -1.24, 0.83 | -0.01 (0.10)  -0.10 (0.08)  -0.36 (0.07)  *Reference*  -0.11 (0.10)  -0.003 (0.09)  *Reference*  -0.31 (0.42) | 0.93  0.25  <0.01  --  0.28  0.97  --  0.46 | -0.20, 0.18  -0.26, 0.07  -0.49, -0.22  --  -0.30, 0.09  -0.18, 0.17  --  -1.14, 0.51 |
| *Adjusted cases compared to controls at single time-points* | T1  T2  T3 | 0.20 (0.10)  0.19 (0.10)  0.25 (0.10) | 0.04  0.06  0.01 | 0.01, 0.39  -0.01, 0.38  0.05, 0.45 | -0.11 (0.09)  -0.01 (0.09)  -0.01 (0.10) | 0.22  0.89  0.93 | -0.30, 0.07  -0.18, 0.16  -0.20, 0.18 |
| **DHEAS**  **(µmol/l)** | Case  T1  T2  T3  Case#T1  Case#T2  Case#T3  Constant | -0.08 (0.11)  0.03 (0.08)  -0.08 (0.05)  *Reference*  0.07 (0.08)  0.05 (0.06)  *Reference*  9.87 (0.39) | 0.49  0.74  0.09  --  0.40  0.39  --  <0.01 | -0.29, 0.14  -0.13, 0.18  -0.18, 0.01  --  -0.09, 0.22  -0.07, 0.18  --  9.11, 10.6 | 0.09 (0.10)  -0.21 (0.09)  -0.19 (0.05)  *Reference*  0.03 (0.08)  0.06 (0.08)  *Reference*  2.24 (0.46) | 0.40  0.01  <0.01  --  0.71  0.45  --  <0.01 | -0.12, 0.29  -0.38, -0.05  -0.28, -0.09  --  -0.13, 0.20  -0.09, 0.20  --  1.34, 3.14 |
| *Adjusted cases compared to controls at single time-points* | T1  T2  T3 | -0.01 (0.08)  -0.02 (0.09)  -0.08 (0.11) | 0.89  0.80  0.49 | -0.17, 0.15  -0.19, 0.15  -0.29, 0.14 | 0.12 (0.09)  0.14 (0.09)  0.09 (0.10) | 0.17  012  0.40 | -0.05, 0.29  -0.04, 0.32  -0.12, 0.29 |
| **11KT (nmol/l)** | Case  T1  T2  T3  Case#T1  Case#T2  Case#T3  Constant | 0.22 (0.09)  0.06 (0.10)  -0.14 (0.06)  *Reference*  -0.03 (0.10)  -0.03 (0.08)  *Reference*  -0.42 (0.38) | 0.02  0.58  0.03  --  0.79  0.70  --  0.27 | 0.04, 0.41  -0.14, 0.25  -0.26, -0.01  --  -0.22, 0.17  -0.19, 0.13  --  -1.16, 0.33 | 0.08 (0.10)  0.10 (0.09)  -0.06 (0.06)  *Reference*  -0.16 (0.09)  0.002 (0.09)  *Reference*  -0.33 (0.46) | 0.43  0.25  0.35  --  0.08  0.98  --  0.47 | -0.11, 0.27  -0.07, 0.28  -0.18, 0.06  --  -0.34, 0.02  -0.17, 0.17  --  -1.24, 0.57 |
| *Adjusted cases compared to controls at single time-points* | T1  T2  T3 | 0.19 (0.10)  0.19 (0.10)  0.22 (0.09) | 0.04  0.05  0.02 | 0.01, 0.38  0.002, 0.38  0.04, 0.41 | -0.08 (0.09)  0.08 (0.09)  0.08 (0.10) | 0.35  0.40  0.43 | -0.26, 0.09  -0.10, 0.26  -0.11, 0.27 |
| **Testosterone**  **(nmol/l)** | Case  T1  T2  T3  Case#T1  Case#T2  Case#T3  Constant | -0.11 (0.06)  0.02 (0.07)  0.02 (0.04)  *Reference*  0.02 (0.06)  -0.002 (0.06)  *Reference*  3.94 (0.36) | 0.09  0.81  0.65  --  0.80  0.98  --  <0.01 | -0.23, 0.02  -0.12, 0.15  -0.07, 0.11  --  -0.10, 0.13  -0.12, 0.11  --  3.24, 4.64 | -0.05 (0.10)  -0.02 (0.10)  0.45 (0.31)  *Reference*  -0.07 (0.10)  -0.43 (0.28)  *Reference*  -1.47 (0.71) | 0.63  0.81  0.14  --  0.52  0.12  --  0.04 | -0.24, 0.14  -0.22, 0.17  -0.15, 1.05  --  -0.27, 0.14  -0.97, 0.11  --  -2.86, -0.09 |
| *Adjusted cases compared to controls at single time-points* | T1  T2  T3 | -0.09 (0.06)  -0.11 (0.06)  -0.11 (0.06) | 0.15  0.09  0.09 | -0.22, 0.03  -0.23, 0.02  -0.23, 0.02 | -0.11 (0.08)  -0.47 (0.31)  0.05 (0.10) | 0.15  0.13  0.63 | -0.27, 0.04  -1.08, 0.14  -0.24, 0.14 |

^a^Adjusted for age, body mass index, and physical activity, and additionally for age at menarche, parity, menstrual status, and use of oral/intrauterine contraceptives for women.

Sample numbers at each time point: women: 60 cases and 75 controls; men: 56 cases and 63 controls. 11KA4, 11-ketoandrostenedione; 11KT, 11-ketotestosterone; 11OHT, 11-hydroxytestosterone; 11OHA4, 11-hydroxyandrostenedione; A4, androstenedione; DHEAS, dehydroepiandrosterone; SE, standard error; T1, Tromsø3 (1986/87); T2, Tromsø4 (1994/95); T3, Tromsø5 (2001).

Table S5. Crude and adjusted^a^ ORs of type 2 diabetes for each androgen by time-point and sex. The Tromsø Study 1986– 2001.

|  |  | **T1 (1986/87)** | | **T2 (1994/95)** | | **T3 (2001)** | |
| --- | --- | --- | --- | --- | --- | --- | --- |
|  |  | **Crude** | **Adjusted^a^** | **Crude** | **Adjusted^a^** | **Crude** | **Adjusted^a^** |
| **Biomarker** | **Sex** | **OR**  **(95%CI)** | **OR**  **(95%CI)** | **OR**  **(95%CI)** | **OR**  **(95%CI)** | **OR**  **(95%CI)** | **OR**  **(95%CI)** |
| **11OHT (nmol/l)** | Women  Men | 1.16  (0.75, 1.78)  1.63  (1.12, 2.38) | 0.70  (0.41, 1.20)  1.42  (0.92, 2.17) | 2.20  (1.34, 3.63)  2.09  (1.32, 3.32) | 1.67  (0.80, 3.46)  1.56  (0.99, 2.44) | 1.61  (1.11, 2.34)  2.16  (1.40, 3.32) | 1.23  (0.78, 1.93)  1.76  (1.12, 2.75) |
| **11OHA4 (nmol/l)** | Women  Men | 0.97  (0.62, 1.52)  1.36  (0.94, 1.97) | 0.78  (0.44, 1.38)  1.30  (0.86, 1.97) | 1.63  (0.99, 2.68)  1.83  (1.11, 3.04) | 1.39  (0.62, 3.13)  1.57  (0.90, 2.72) | 1.34  (0.89, 2.02)  2.25  (1.33, 3.82) | 1.03  (0.60, 1.76)  2.00  (1.12, 3.56) |
| **A4**  **(nmol/l)** | Women  Men | 0.84  (0.54, 1.31)  0.90  (0.69, 1.18) | 1.02  (0.52, 1.97)  0.92  (0.66, 1.27) | 0.89  (0.50, 1.57)  0.80  (0.51, 1.28) | 1.35  (0.47, 3.90)  1.00  (0.56, 1.78) | 0.99  (0.58, 1.70)  0.92  (0.60, 1.40) | 0.99  (0.47, 2.10)  1.00  (0.61, 1.63) |
| **11KA4 (nmol/l)** | Women  Men | 0.87  (0.56, 1.34)  1.52  (1.03, 2.24) | 0.71  (0.41, 1.22)  1.59  (1.02, 2.49) | 1.28  (0.72, 2.25)  1.54  (0.90, 2.61) | 0.92  (0.36, 2.39)  1.64  (0.92, 2.92) | 1.21  (0.82, 1.77)  2.03  (1.26, 3.27) | 0.92  (0.55, 1.53)  1.84  (1.09, 3.10) |
| **DHEAS**  **(µmol/l)** | Women  Men | 1.00  (0.67, 1.45)  0.80  (0.46, 1.42) | 1.35  (0.76, 2.42)  0.95  (0.45, 2.04) | 1.07  (0.69, 1.65)  0.72  (0.38, 1.36) | 1.94  (0.79, 4.76)  0.83  (0.35, 1.97) | 0.93  (0.61, 1.44)  0.63  (0.33, 1.22) | 1.22  (0.68, 2.20)  0.65  (0.29, 1.46) |
| **11KT (nmol/l)** | Women  Men | 1.04  (0.68, 1.58)  1.85  (1.24, 2.78) | 0.74  (0.43, 1.28)  1.62  (1.02, 2.57) | 1.63  (1.01, 2.63)  2.15  (1.28, 3.61) | 1.23  (0.60, 2.53)  1.64  (0.94, 2.86) | 1.52  (1.02, 2.25)  2.19  (1.36, 3.51) | 1.30  (0.80, 2.10)  1.78  (1.03, 3.08) |
| **Testosteron**  **(nmol/l)** | Women  Men | 0.86  (0.54, 1.39)  0.41  (0.23, 0.75) | 0.83  (0.44, 1.58)  0.60  (0.31, 1.19) | 0.99  (0.93, 1.06)  0.36  (0.19, 0.67) | 0.65  (0.33, 1.29)  0.52  (0.25, 1.08) | 1.14  (0.78, 1.68)  0.32  (0.16, 0.63) | 0.99  (0.62, 1.60)  0.45  (0.21, 0.98) |

^a^Adjusted for age, body mass index, and physical activity, and additionally age at menarche, parity, menstrual status, and use of oral/intrauterine contraceptives for women.

Odds ratios for all androgens are estimated per 1-IQR increase. Sample numbers at each time point: women: 60 cases and 75 controls; men: 56 cases and 63 controls. 11KA4, 11-ketoandrostenedione; 11KT, 11-ketotestosterone; 11OHT, 11-hydroxytestosterone; 11OHA4, 11-hydroxyandrostenedione; A4, androstenedione; CI, confidence interval; DHEAS, dehydroepiandrosterone sulphate; IQR, interquartile range; OR, odds ratio; T1, Tromsø3 (1986/87); T2, Tromsø4 (1994/95); T3, Tromsø5 (2001).
